# Niclosamide Prevents the Formation of Large Ubiquitin-Containing Aggregates Caused by Proteasome Inhibition

**DOI:** 10.1371/journal.pone.0014410

**Published:** 2010-12-23

**Authors:** Esther Gies, Inga Wilde, Jason M. Winget, Maria Brack, Barak Rotblat, Carolina Arias Novoa, Aruna D. Balgi, Poul H. Sorensen, Michel Roberge, Thibault Mayor

**Affiliations:** 1 Department of Biochemistry and Molecular Biology, University of British Columbia, Vancouver, British Columbia, Canada; 2 Centre for High-Throughput Biology, University of British Columbia, Vancouver, British Columbia, Canada; 3 Department of Molecular Oncology, British Columbia Cancer Research Centre, Vancouver, British Columbia, Canada; 4 Department of Pathology, University of British Columbia, Vancouver, British Columbia, Canada; University of North Dakota, United States of America

## Abstract

**Background:**

Protein aggregation is a hallmark of many neurodegenerative diseases and has been linked to the failure to degrade misfolded and damaged proteins. In the cell, aberrant proteins are degraded by the ubiquitin proteasome system that mainly targets short-lived proteins, or by the lysosomes that mostly clear long-lived and poorly soluble proteins. Both systems are interconnected and, in some instances, autophagy can redirect proteasome substrates to the lysosomes.

**Principal Findings:**

To better understand the interplay between these two systems, we established a neuroblastoma cell population stably expressing the GFP-ubiquitin fusion protein. We show that inhibition of the proteasome leads to the formation of large ubiquitin-containing inclusions accompanied by lower solubility of the ubiquitin conjugates. Strikingly, the formation of the ubiquitin-containing aggregates does not require ectopic expression of disease-specific proteins. Moreover, formation of these focused inclusions caused by proteasome inhibition requires the lysine 63 (K63) of ubiquitin. We then assessed selected compounds that stimulate autophagy and found that the antihelmintic chemical niclosamide prevents large aggregate formation induced by proteasome inhibition, while the prototypical mTORC1 inhibitor rapamycin had no apparent effect. Niclosamide also precludes the accumulation of poly-ubiquitinated proteins and of p62 upon proteasome inhibition. Moreover, niclosamide induces a change in lysosome distribution in the cell that, in the absence of proteasome activity, may favor the uptake into lysosomes of ubiquitinated proteins before they form large aggregates.

**Conclusions:**

Our results indicate that proteasome inhibition provokes the formation of large ubiquitin containing aggregates in tissue culture cells, even in the absence of disease specific proteins. Furthermore our study suggests that the autophagy-inducing compound niclosamide may promote the selective clearance of ubiquitinated proteins in the absence of proteasome activity.

## Introduction

Protein homeostasis, which maintains the balance between protein synthesis, folding and clearance, is central to cell survival. The ubiquitin proteasome system (UPS) plays a major role in this process by selectively degrading a large portion of short-lived proteins [1]. For instance, under some conditions, up to 30% of newly synthesized proteins are directly eliminated by the UPS [2]. Selective degradation by the UPS is a two-step process, in which the substrate is first covalently attached to ubiquitin, a small 76-amino-acid protein, and then targeted to the large multimeric proteasome complex for proteolysis. Ubiquitin conjugation relies on an enzymatic cascade driven by the E1 activating, E2 conjugating and E3 ligase enzymes [3]. In the human genome, an estimated six hundred genes encode putative E3s [4]. Beside proteolysis, ubiquitination regulates many other processes such as endocytosis and chromatin remodelling [5]. To be efficiently targeted to the proteasome, substrate proteins have to be attached to a poly-ubiquitin chain, in which at least four ubiquitin molecules are successively linked through specific lysine residues of ubiquitin (e.g., K48 and K11) [6], [7]. Prior to proteolysis, ubiquitin itself is removed from the substrate by deubiquitinating enzymes and recycled in the cell. The UPS degrades both key regulatory proteins (e.g., cell cycle regulators), as well as misfolded and damaged proteins. Failure to degrade misfolded proteins leads to their accumulation and aggregation in the cell.

Protein aggregation is also a hallmark of a large number of age-related neurodegenerative pathologies [8], [9]. Aggregation may be induced by the extended exposure of misfolded domains and non-specific hydrophobic interactions that result in the formation of amorphous structures [10]. Alternatively, aggregation can be induced by highly ordered β–strand fibrils that form insoluble amyloids. Mutations or modifications in disease-specific proteins can cause misfolding and consequently formation of aggregates. For example, polyglutamine repeat expansion in huntingtin triggers the formation of insoluble amyloid-like structures in Huntington's disease [11]. Misfolded proteins first form small proto-fibrils or inclusions and then are sequestered into larger aggregates by microtubule-mediated retrograde transport [12]. A major view is that aggregation may prevent cytotoxicity by shielding abnormal proteins from non-specific interactions with other proteins [13], [14], [15].

Impairment of the UPS has been linked to protein aggregation [16], [17]. Evidence for this view is that ubiquitin is enriched in most symptomatic aggregates, and chemical inhibition of the proteasome induces the formation of aggregates similar to those found in several diseases [18], [19], [20], [21]. Proteasome inhibition also accelerates or promotes the aggregation of ectopically expressed disease-specific proteins like huntingtin and α-synuclein [11], [22].

Clearance of insoluble proteins by the lysosomes can be mediated by macro-autophagy (hereafter called autophagy). This pathway is characterized by the formation of double-membraned vesicles that engulf cytoplasmic materials, including large protein complexes, followed by fusion with lysosomes and digestion by acidic hydrolases [23]. Long-lived and damaged proteins that are poorly soluble are thought to be mainly degraded by the lysosomes, as the proteasome cannot efficiently process them [24]. Induction of autophagy has been shown to reduce the aggregation of disease-specific proteins [25], [26], [27]. The UPS and autophagy pathways are interrelated [24] and both critically influence events associated with aggregation [28], [29].

In the present study, we used neuroblastoma cells that stably express GFP-ubiquitin to show that ubiquitin-containing aggregates can be readily and quantitatively monitored upon chemical inhibition of the proteasome. We found that the formation of these ubiquitin-containing inclusions required K63 of ubiquitin, which has been previously linked to clearance of ubiquitinated proteins by the authophagy pathway [30], [31]. Then we tested several recently identified autophagy-modulating compounds and found that the antihelmintic chemical niclosamide prevented the formation of large ubiquitin-containing aggregates. Niclosamide also affected the distribution of lysosomes in the cell. These results suggest that niclosamide may induce the clearance of ubiquitinated proteins that accumulate during proteasome inhibition.

## Results

### Proteasome inhibition induces the formation of cellular inclusions containing GFP-ubiquitin

We sought to monitor the formation of ubiquitin-containing protein aggregates after inhibition of the proteasome. We used green fluorescent protein (GFP) N-terminally fused to ubiquitin, which produces a functional tagged ubiquitin (i.e., that can be conjugated) [32]. Upon transient transfection in SH-SY5Y neuronal cells, GFP-ubiquitin positive inclusions were formed after 8 h incubation with the proteasome inhibitor MG132 (Fig. S1A). No inclusions were detected when transiently transfected cells were treated with DMSO or when MG132 was applied to cells expressing GFP alone (Fig. S1A). In wild-type MG132-treated cells, we also directly observed ubiquitin-containing aggregates by immunofluorescence (data not shown). However, due to high background fluorescence, it was difficult to quantify these aggregates.

**Figure 1:**
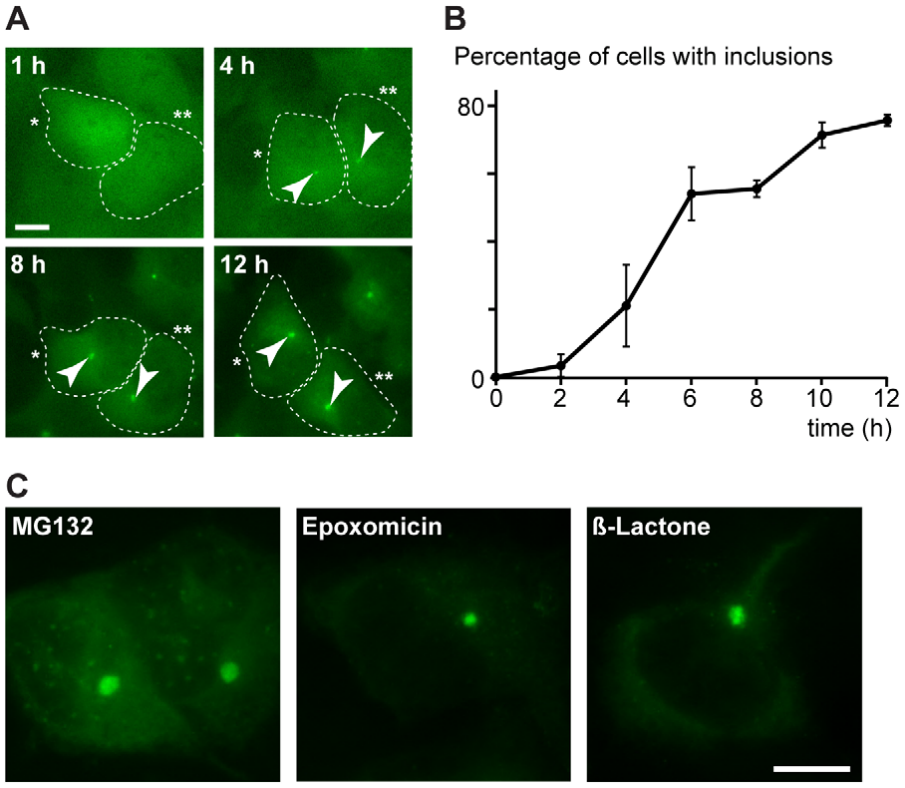
Ubiquitin-containing aggregates caused by proteasome inhibition. (**A**) Formation of aggregates (arrowheads) induced with 20 µM MG132 was monitored by live-imaging a SH-SY5Y cell population that stably expresses GFP-ubiquitin. Two representative cells (indicated with one and two asterisks) are shown at different times. Scale bar represents 10 µm. (**B**) Percentage of methanol-fixed GFP-ubiquitin SH-SY5Y cells containing at least one GFP-ubiquitin inclusion (defined by a four fold increase of GFP signal intensity) after the addition of 20 µM MG132 for the indicated times. Average of three experiments with standard errors are shown (n = 200). (**C**) Representative methanol-fixed GFP-ubiquitin cells treated with 10 µM MG132, 1 µM epoxomycin or 2 µM clasto-lactacystin β-lactone for 12 h. Scale bar represents 10 µm.

We subsequently established a cell population that stably expresses GFP-ubiquitin and performed time-lapse microscopy experiments to monitor the formation of the inclusions. Following the addition of MG132, we detected a small punctum near the nucleus in many cells after 4 h, which further increased in size over time (Fig. 1A, Sup. Movie S1). We confirmed this observation by analyzing fixed cells, and found that GFP-ubiquitin containing inclusions were present in up to 80% of the cells after 12 h of MG132 treatment (Fig. 1B), while mock treatment did not affect GFP-ubiquitin distribution (data not shown). Large inclusions were observed after the addition of 5 to 20 µM MG132 for 8 to 12 h. Typically, cells contained one large inclusion with additional smaller inclusions in few cases (Fig. S1B). Ubiquitin was also directly detected by immunofluorescence in these inclusions (Fig. S1C). In addition, incubation of the GFP-ubiquitin expressing cells with epoxomicin or clasto-lactacystin β-lactone, which are specific irreversible inhibitors of the proteasome [33], [34], induced GFP-ubiquitin enriched inclusions similar to those seen during MG132 treatment (Fig. 1C).

### The MG132-induced inclusions contained GFP-ubiquitin conjugated to low solubility polypeptides

**Figure 2:**
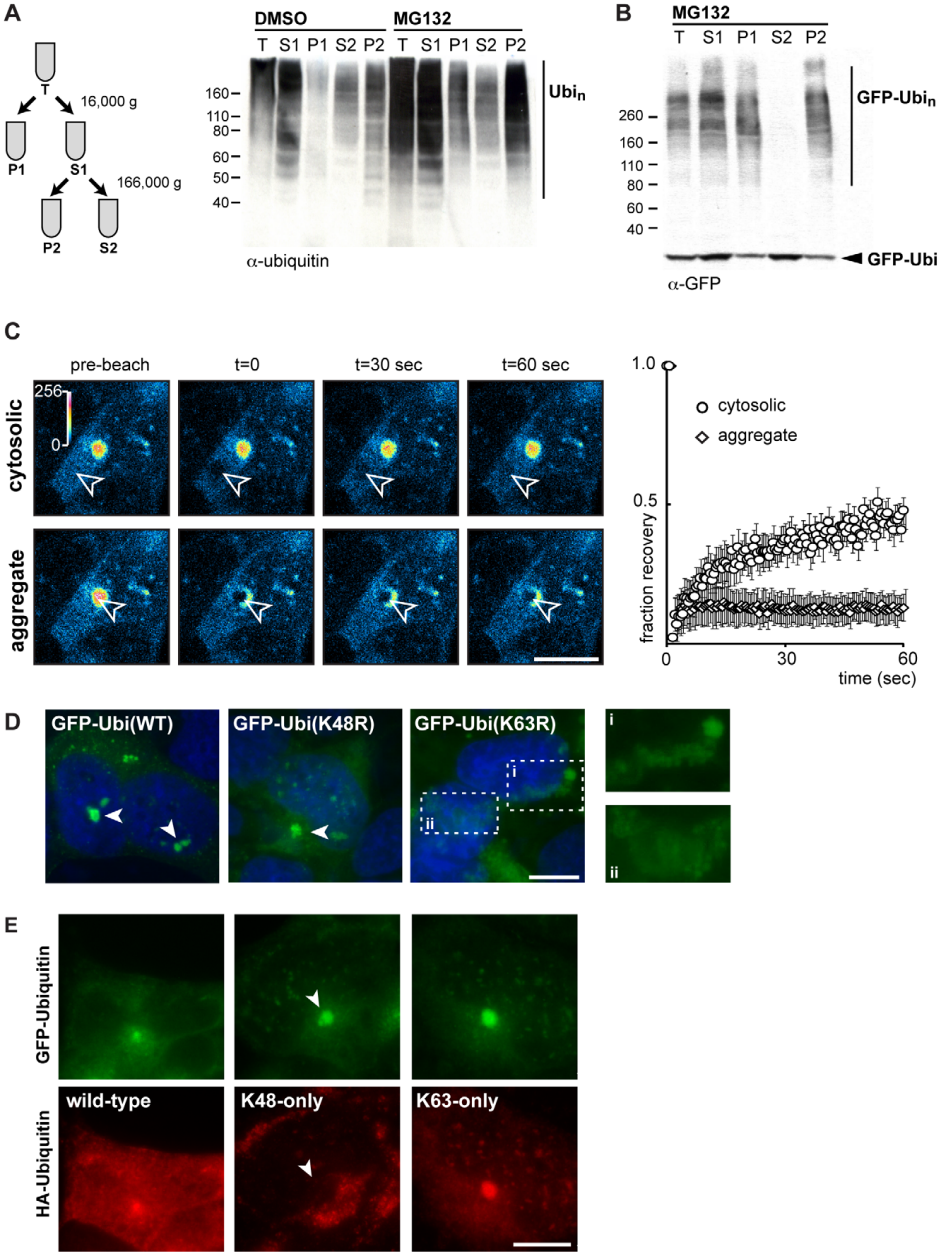
Low solubility proteasome substrates aggregate in a ubiquitin K63-dependent manner. (**A**) Schematic diagram of the cell fractionation based on protein solubility (left). Wild-type SH-SY5Y cells were incubated with 20 µM MG132 or DMSO for 12 h prior to lysis with 0.5% NP40 buffer. Total cell lysates (T), detergent-soluble (S1) and insoluble (P1) fractions, and high-speed soluble (S2) and insoluble (P2) fractions were subjected to 4–20% SDS-PAGE and immunoblotting with an anti-ubiquitin antibody. (**B**) GFP-ubiquitin SH-SY5Y cells were treated with 20 µM MG132 for 12 h and analyzed as in A. (**C**) GFP-ubiquitin SHY5Y cells treated with 10 µM MG132 for 12 hours were imaged live by fluorescence confocal microscopy before and after photobleaching of on area in the cytoplasm (top) or in the inclusion (bottom). Typical images of SH-Y5Y GFP-ubiquitin cells before, immediately after and 30 and 60 seconds after photobleaching are shown. Color-coded pixel fluorescence intensities are shown in the right panel. Fluorescence intensities relative to the pre-treated area were reported for each time point (right). Data represent the mean ± SEM (n = 7 cytoplasm; n = 4 inclusions) of the normalized bleached region. (**D**) SH-SY5Y cells transiently transfected with the indicated GFP-ubiquitin constructs for 24 h prior to treatment with 5 µM MG132 for 8 h and PFA fixation with Hoechst. Two insets showing cells transfected with K63R-ubiquitin are shown to the right. (**E**) GFP-ubiquitin SH-SY5Y cells transiently transfected with the indicated HA-ubiquitin constructs for 24 h prior to treatment with 5 µM MG132 for 12 h and methanol fixation. Immuno-fluorescence was performed using mouse anti-HA and anti-mouse Alexa568 (Invitrogen) antibodies. All scale bars represent 10 µm.

We reasoned that the MG132-induced inclusions might be composed of misfolded and aggregate-prone proteins that were no longer degraded after proteasome inhibition. In order to test this idea, we examined whether the ubiquitinated proteins that accumulate upon proteasome inhibition displayed a lower solubility. We first examined ubiquitinated proteins in wild-type SH-SY5Y cells to avoid any effect of GFP on solubility. After clearing cell lysates by centrifugation (S1), we performed a high-speed centrifugation at 166,000 g and found that the large majority of ubiquitinated proteins that accumulated during the MG132 treatment were not soluble (P2; Fig. 2A). A similar solubility profile was observed when polypeptides were conjugated to the GFP-ubiquitin fusion (Fig. 2B). Remarkably, while the majority of unconjugated GFP-ubiquitin remained in the soluble fraction, the low solubility fraction was mainly enriched with conjugated GFP-ubiquitin. This data strongly suggests that the inclusions observed in MG132-treated cells were composed of GFP-ubiquitin conjugated to low solubility proteins.

We next sought to determine whether ubiquitinated proteins were persistent in the MG132-induced inclusions. It was previously shown that aggregating proteins have low exchange rates with their cytoplasmic pools [35], [36]. In contrast, proteins involved in the aggregation process display a more rapid dynamics and shuttle from the aggregates to the cytosol. For instance, the pool of hsp70 in huntingtin aggregates is replaced by cytosolic hsp70 within less then one minute [36]. To assess the diffusion or exchange rates of proteins conjugated to GFP-ubiquitin in the MG132-induced inclusions, we performed fluorescence recovery after photo-bleaching (FRAP). While the cytosolic GFP-ubiquitin signal was rapidly recovered in a photo-bleached area, the loss of fluorescence lasted over one minute in the ubiquitin-containing inclusions after FRAP (Fig. 2C). This persistent loss suggested that ubiquitinated proteins were stable in the aggregates and not rapidly exchanged with other ubiquitinated cytosolic proteins. We conclude that the GFP-ubiquitin containing inclusions caused by proteasome inhibition were likely composed of non-degraded and aggregation-prone proteins conjugated to GFP-ubiquitin. We hereafter refer to these structures as aggregates. Notably, these ubiquitin-containing aggregates were formed in the absence of ectopic expression of disease-related proteins prone to aggregation.

### Formation of ubiquitin containing aggregates requires K63 conjugation

It was previously suggested that formation of aggregates in several models requires formation of poly-ubiquitin chains linked by lysine 63 (K63) [30], [31], [37], [38]. To determine whether specific types of ubiquitin chains are associated with the formation of ubiquitin-containing aggregates caused by proteasome inhibition, we transfected different GFP-ubiquitin mutants in SH-SY5Y cells. Formation of aggregates was similar between wild-type ubiquitin and the K48R-ubiquitin mutant (in which the K48 was mutated to a arginine residue; Fig. 2D). In contrast, the K63R-ubiquitin mutant was unable to form focused aggregates after proteasome inhibition, and instead, led to the accumulation of punctuate-like structures in the periphery of the cytoplasm (Fig. 2D; see insets). This data suggests that conjugation of K63 is required for the formation of large and focused ubiquitin containing aggregates. To further test the requirement of K63 linkage in the aggregation of proteasome substrates, we transfected additional ubiquitin mutants tagged to HA (hemagglutinin) in SH-SY5Y cells that stably expressed GFP-ubiquitin. We found that the K63-only ubiquitin mutant (in which all K except K63 are mutated to R) formed a normal aggregate after MG132 treatment (Fig. 2E). This data indicates that K63 is sufficient for the formation of induced aggregates. Remarkably, the K48-only ubiquitin mutant was unable to form aggregates, in agreement with a requirement for K63 (Fig. 2E). All together, these results indicate that ubiquitinated proteins that localize in the aggregates after proteasome inhibition are conjugated to poly-ubiquitin chains principally linked by K63.

### Formation of large ubiquitin-containing aggregates requires an intact microtubule network

**Figure 3:**
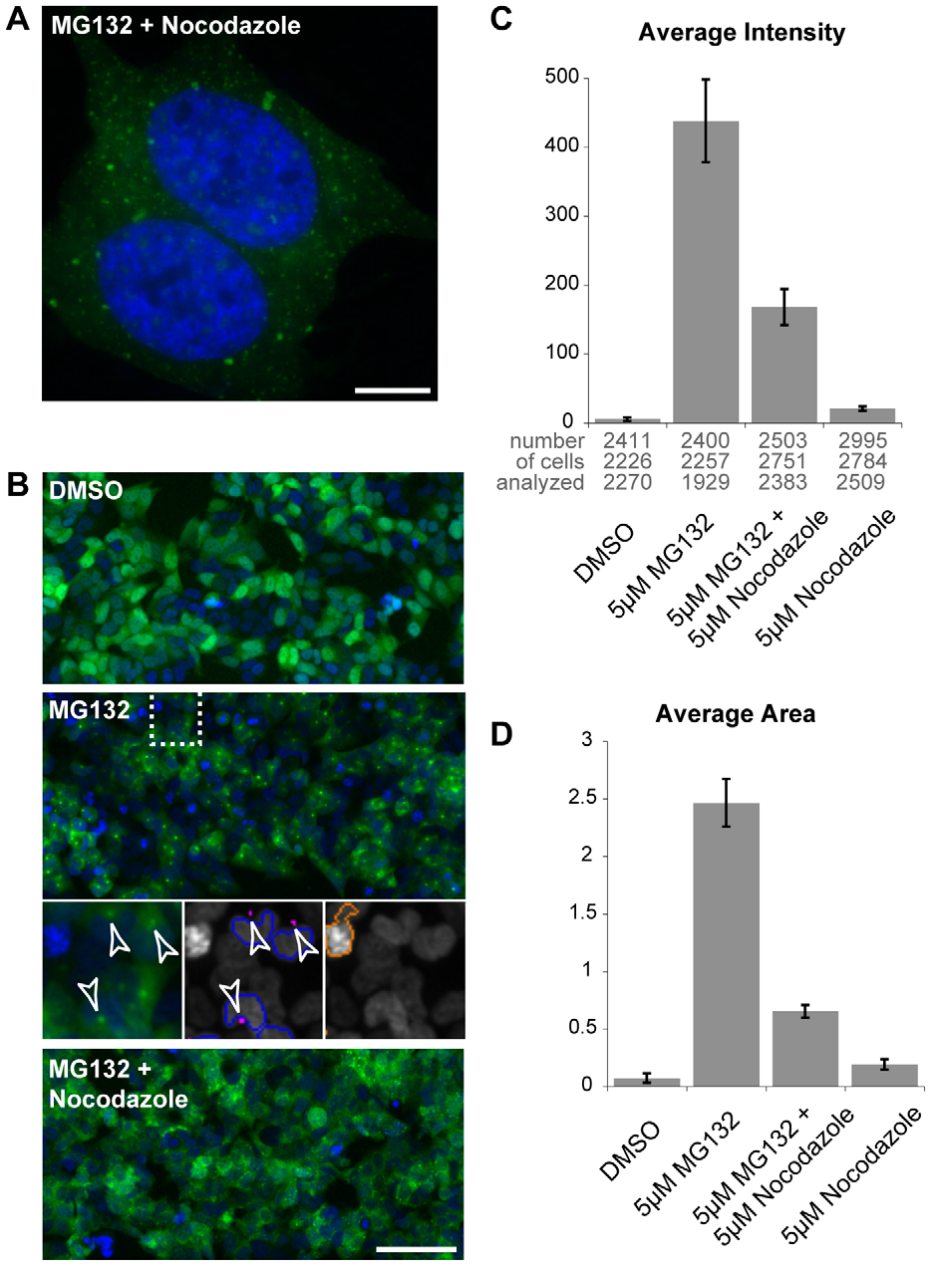
Formation of induced GFP-ubiquitin aggregates is impaired by nocodazole. (**A**) GFP-ubiquitin SH-SY5Y cells were treated with 5 µM MG132 together with 2 µM nocodazole for 8 h prior to methanol fixation and Hoechst staining (blue). Scale bar represents 10 µm. (**B**, **C** and **D**) GFP-ubiquitin SH-SY5Y cells were treated as indicated with 5 µM MG132 and 5 µM nocodazole for 8 h prior to fixation and quantification with the automated high-content fluorescence imager. Representative images are shown (**B**). Insets show MG132-treated cells detected with a Hoechst mask (blue contours) and their associated aggregates (purple; arrowheads), as well as an object rejected (red contour) because of its strong signal intensity. Scale bar represents 100 µm. Signal intensity (**C**) and area in µm^2^ (**D**) of the GFP-ubiquitin aggregates. Results are the mean of three independent wells with standard deviations. The numbers of analyzed cells to calculate the average intensity (or area) in each well are indicated below the histogram.

The microtubule network is required for the formation of large aggresomes, as smaller aggregates or proto-fibrils are retrogradely transported by dynein motor proteins to the centrosome [39], [40], [41]. We found that the MG132-induced inclusions enriched with GFP-ubiquitin co-localized with γ-tubulin, which primarily localizes to the centrosome (Fig. S2A). Moreover, the addition of the microtubule-depolarizing agent nocodazole together with MG132 induced a distinct pattern with multiple small GFP-ubiquitin puncta spread throughout the cytoplasm (Fig. 3A, S2B). Similar results were obtained when nocodazole was added together with clasto-lactacystin β-lactone (Fig. S2B). To better quantify this phenomenon, we analyzed the data using an automated high-content fluorescence imager. Individual cells were detected after applying a mask around Hoechst-stained nuclei, and induced GFP-ubiquitin aggregates were identified using a GFP-signal intensity threshold (Fig. 3B; see purple puncta in middle inset). After the addition of MG132, the induced aggregates were detected as a strong increase in signal intensity (Fig. 3C). The addition of nocodazole induced a major reduction in both aggregate average intensity signal (2.6 fold, Fig. 3C) and aggregate average size (3.8 fold, Fig. 3D). This decrease was caused by the reduction of the GFP-signal intensity below the threshold in most inclusions, and confirmed that depolymerisation of microtubules prevented the formation of large aggregates in the cell. Thus, the large GFP-ubiquitin inclusions induced by proteasome inhibition were localized next to or around the centrosome and required an intact microtubule network for their formation. Overall, these data indicate that the aggregates are similar to previously observed aggresome structures [42].

### Niclosamide inhibits the formation of the large ubiquitin-containing inclusions induced after proteasome inhibition

**Figure 4:**
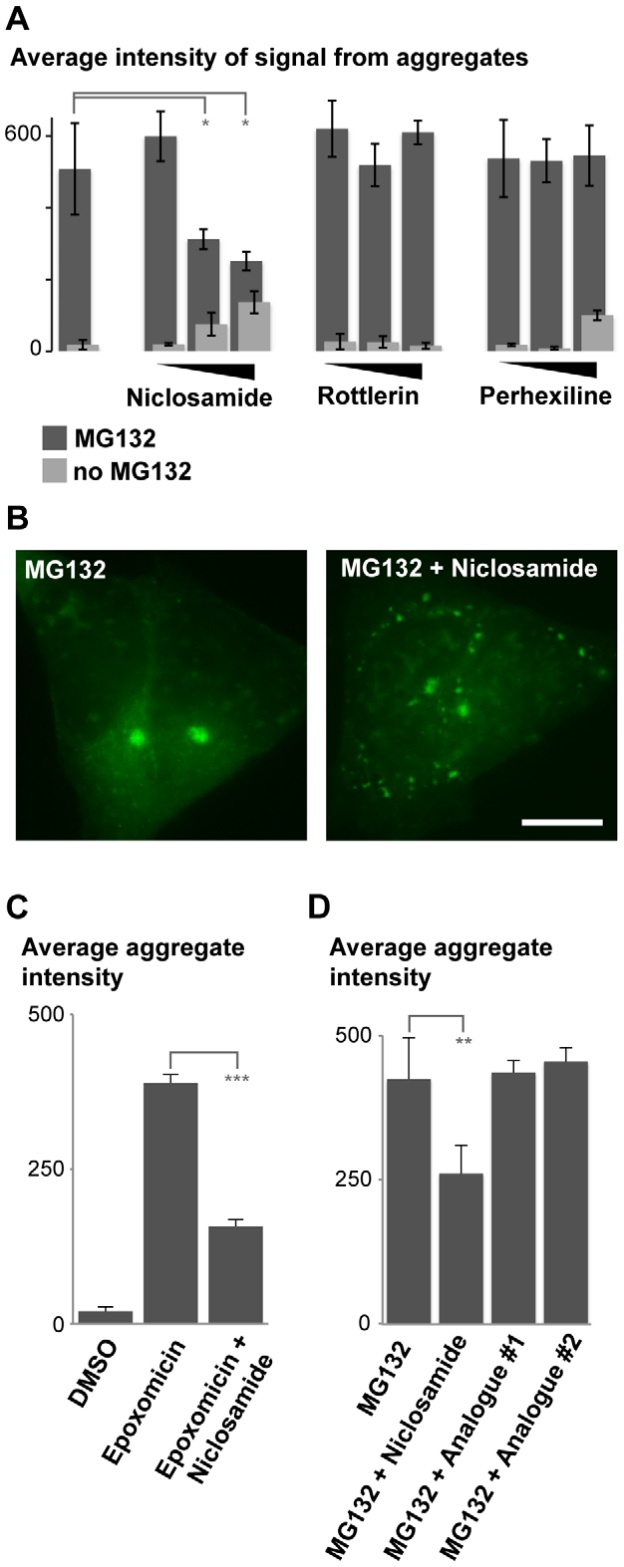
Niclosamide affects the formation of the ubiquitin-enriched aggregates caused by proteasome inhibition in SH-SY5Y cells. (**A**) Signal intensities of the aggregates in SH-SY5Y cells stably expressing GFP-ubiquitin treated for 8 h with different autophagy inducers. The following concentrations were used: niclosamide (1, 3 and 10 µM), rottlerin (0.1, 1, 3 µM) and perhexiline (1, 3, 10 µM) alone (light grey), and together with 5 µM MG132 (dark grey). Mean intensities (with standard deviations) were measured in three independent wells for each condition using the automated high-content fluorescence imager. The p values for the designated samples calculated by Student's *t*-test were 0.03 and 0.0014, respectively. (**B**) Representative images of cells incubated as indicated with 5 µM MG132 and 10 µM niclosamide for 8 h prior to PFA fixation. Scale bar represents 10 µm. Preceding time points are shown in Fig. S3C. (**C**, **D**) Same as in A, using 1 µM epoxomycin, 5 µM MG132, 10 µM niclosamide or 10 µM of niclosamide analogues #1 and #2. The p values for the designated samples calculated by Student's *t*-test were <0.0001 (C) and 0.006 (D).

Autophagy has been shown to participate in clearing cellular aggregates formed by disease-specific proteins [25], [26], [27]. We recently identified niclosamide, perhexiline, and rottlerin as compounds capable of rapidly inducing autophagy [43]. We therefore sought to determine whether they can also alter the formation of the ubiquitin-containing aggregates induced by proteasome inhibition. The chemicals were added for 8 h in combination with MG132 to cells stably expressing GFP-ubiquitin, and large aggregates were quantified using the automated high-content imager. Niclosamide, a salicylanilide antihelmintic drug [44], [45], [46] reduced, in a concentration-dependent manner, the induction of large aggregates by MG132 (Fig. 4A, Fig. S4A, B). Treatment with niclosamide led instead to the formation of more but smaller GFP-ubiquitin inclusions (Fig. 4B; Fig. S3C). The two other compounds did not significantly reduce large aggregate accumulation (Fig. 4A). Interestingly, niclosamide alone induced a small increase in signal intensity (Fig. 4A; see below). Maximal response with 5 µM MG132 was reached at 20 µM niclosamide (IC_50_ of ∼5 µM). Moreover, niclosamide reduced the induction of ubiquitin-containing aggregates by epoxomicin (Fig. 4C), arguing against a specific interaction with MG132 itself. Likewise, niclosamide also affected the aggregate size when it was added to cells pre-treated for 4 h with the irreversible proteasome inhibitor epoxomicin. Large ubiquitin-containing aggregates were observed if cells were treated for 4 h with DMSO after washing away epoxomicin, but not when cells were treated with niclosamide (Fig. S3D). In addition, two analogues closely related to niclosamide, which are unable to activate autophagy (Balgi A.D. *et al*., manuscript in preparation), did not affect the formation of the ubiquitin-containing inclusions (Fig. 4D). We deduce that niclosamide is able to specifically alter the formation of the large ubiquitin-containing aggregates caused by proteasome inhibition.

### Niclosamide prevents the accumulation of ubiquitinated proteins after proteasome inhibition and causes a redistribution of the lysosomes in the cell

**Figure 5:**
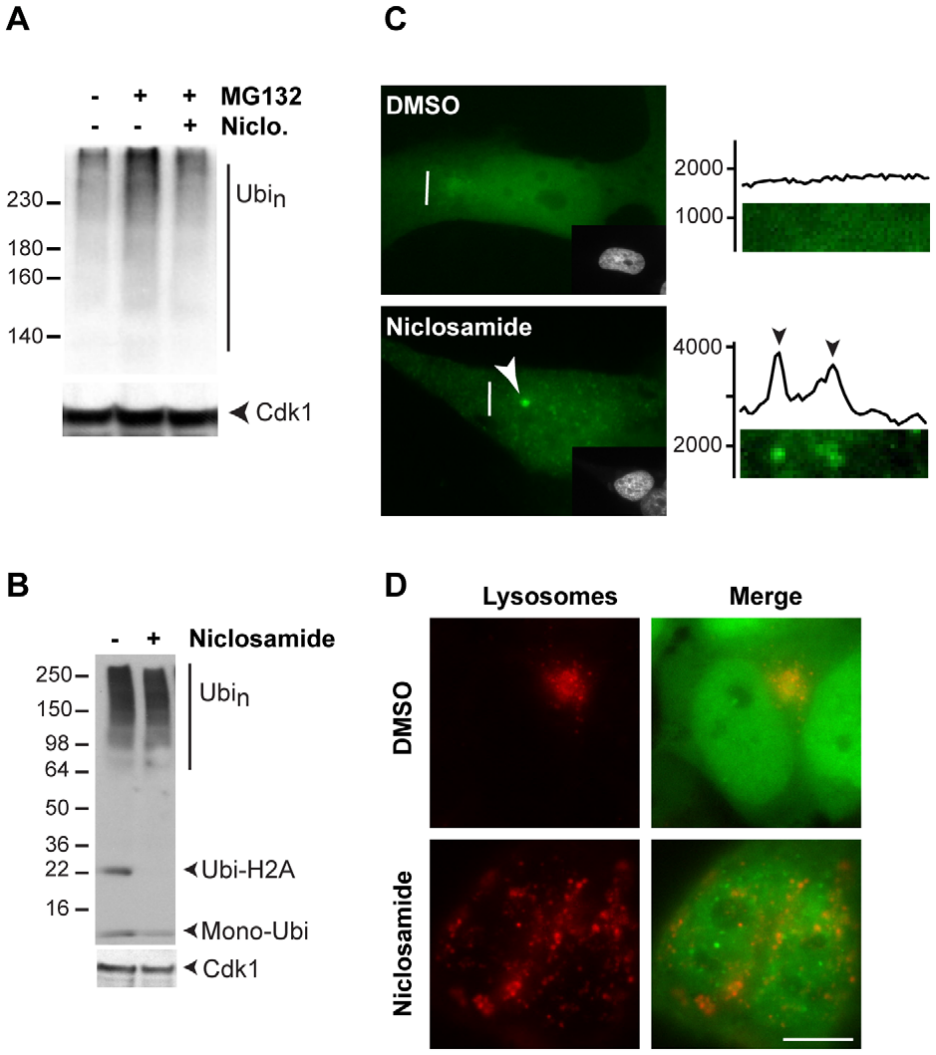
Niclosamide affects ubiquitination levels and lysosome distribution in the cell. (**A**) SH-SY5Y cells were treated as indicated with 5 µM MG132 and 10 µM niclosamide for 8 h prior to lysis in RIPA buffer. Equal amounts of proteins were separated by 4–20% SDS-PAGE followed by immunoblotting with ubiquitin and PSTAIR antibodies. (**B**) Same as A, with cells treated with 10 µM niclosamide or DMSO (0.2%) for 8 h. (**C**) Representative GFP-ubiquitin SH-SY5Y cells treated with 10 µM niclosamide or DMSO for 8 h, prior to PFA fixation and Hoechst staining (shown in the insets). GFP signal based on the camera pixel intensity (vertical axis) for the highlighted 5 µm cross-sections (white bars) is shown on the right side. (**D**) Representative GFP-ubiquitin SH-SY5Y cells treated with 10 µM niclosamide or DMSO for 8 h, as well as with lysotracker for 1 h, prior to imaging of live cells. Scale bar represents 10 µm.

The reduced induction of large ubiquitin-containing aggregates by MG132 in niclosamide-treated cells could be explained by a diminution in the accumulation of ubiquitinated proteins. To test this idea, we first examined the overall ubiquitination levels in SH-SY5Y cells. While treatment with MG132 alone led to an accumulation of ubiquitinated species, addition of niclosamide (with MG132) largely prevented this increase (Fig. 5A). We observed a similar decrease in ubiquitination when we monitored the levels of GFP-ubiquitin conjugation after adding niclosamide and MG132 in cells stably expressing the fusion protein (Fig. S4A). Concurrently, there was no significant increase of ubiquitinated species in the pellets obtained after centrifugation of the cell lysates (data not shown), indicating that the reduction was not due to a change of solubility of the ubiquitin conjugates. One possibility is that niclosamide induces the clearance of ubiquitinated proteins that accumulate after proteasome inhibition. Alternatively, it could prevent the ubiquitination of proteasome substrates.

We next examined whether niclosamide alone could alter ubiquitination levels in cell with normal proteasome activity (i.e., without MG132). While no major change of poly-ubiquitination levels was consistently observed (a minor decrease was observed in some cases), a lower molecular-weight signal (∼22 KDa) that presumably corresponds to ubiquitinated H2A histone protein disappeared in niclosamide-treated cells (Fig. 5B). Ubiquitination is a dynamic process, in which ubiquitinated substrates are in equilibrium between ubiquitination and de-ubiqutination. It has been shown that a reduction in the amount of free mono-ubiquitin in the cell (e.g., due to heat shock stress) causes a decrease in H2A ubiquitination [32], [47]. It was suggested that the enzymes involved in H2A ubiquitination could not compete with other E2/E3s when the amount of free ubiquitin was reduced in the cell, leading to a gradual de-ubiquitination of H2A. We observed that levels of H2A in the cell were not affected by niclosamide (Fig. S4B), suggesting that H2A was likely de-ubiquitinated in the presence of niclosamide. These results indicate that niclosamide may reduce the amount of free ubiquitin in the cell, which would then lead to the de-ubiquitination of H2A.

Niclosamide alone induced a concentration-dependant increase in signal intensity of the GFP-ubiquitin containing aggregates (Fig. 4A). We observed, by microscopy, that a small GFP-ubiquitin positive inclusion was formed next to the nucleus in cells treated with niclosamide alone (Fig. 5C, Fig. S4C; see arrowheads). These inclusions were significantly smaller than the MG312-induced aggregates (see also Fig. S3C). Intriguingly, small puncta were also found throughout the cell, which typically led to a specific increase of GFP-ubiquitin signal intensity (Fig. 5C; see signal quantification of the cross section). For comparison, the GFP-ubiquitin signal remained diffuse in mock-treated cells. These data indicate that niclosamide affects ubiquitin distribution in the cell. Interestingly, niclosamide treatment also induced a change of lysosome distribution in the cell (Fig. 5D; Fig. S4D). In mock-treated cells, lysosomes are primarily found in one region of the cell next to the nucleus. It has been previously shown that lysosomes localize next to the centrosome in a microtubule-dependent manner [48], [49]. Remarkably, lysosomes were redistributed throughout the cell after the addition of niclosamide. Also, the lysosomes appeared larger in niclosamide-treated cells. This data suggests that niclosamide causes a change in lysosome localization and possibly in their activity.

### Inhibition of mTORC1 pathway by rapamycin does not mimic the effect of niclosamide on ubiquitination

**Figure 6:**
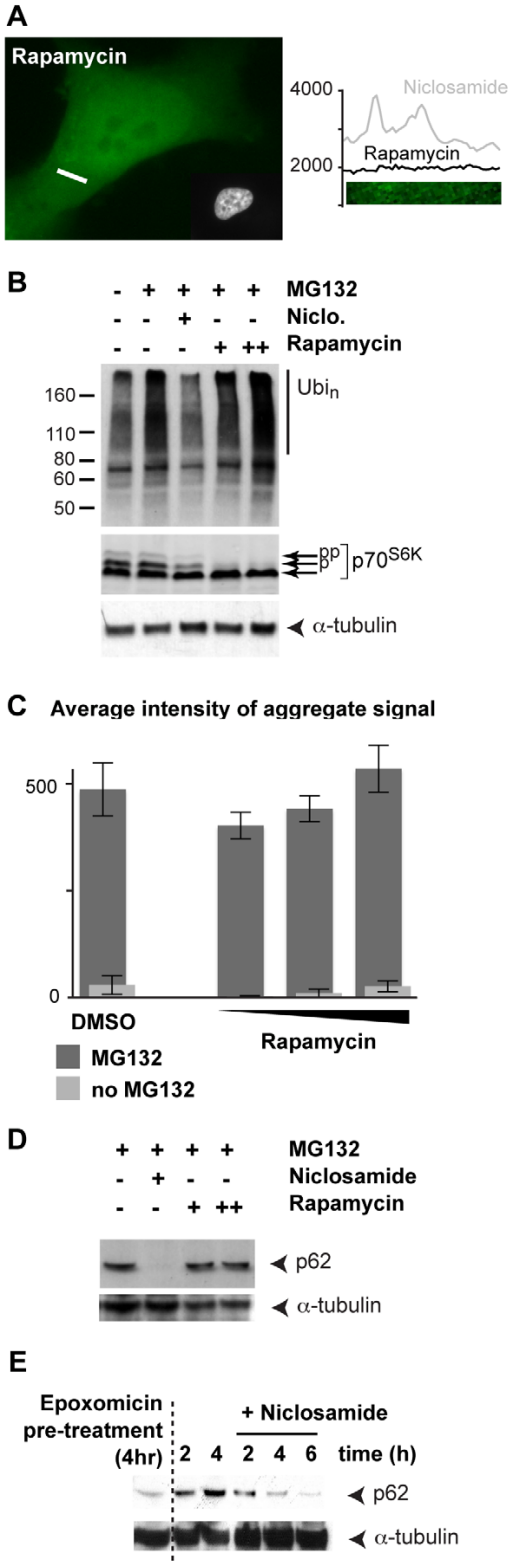
Niclosamide but not rapamycin affects the formation of large ubiquitin-containing aggregates and p62 levels. (**A**) A representative GFP-ubiquitin SH-SY5Y cell treated with 20 nM rapamycin for 8 h, prior to PFA fixation and Hoechst staining. GFP signal intensity (vertical axis) for the indicated 5 µm cross-section (white bar) is shown on the right side. (**B**) SH-SY5Y cells were treated as indicated (5 µM MG132, 10 µM niclosamide, 20 and 100 nM rapamycin) for 8 h, prior to lysis in RIPA buffer. Equal amounts of proteins were separated by 4–20% SDS-PAGE followed by immunoblotting with ubiquitin, SK6 and anti-α-tubulin antibodies. (**C**) Signal intensities of the GFP-ubiquitin aggregates were measured in three independent wells for a each condition using the automated high-content fluorescence imager. Cells were treated with rapamycin (10, 30, 100 nM) alone (light grey), and together with 5 µM MG132 (dark grey) for 8 h. (**D**) Same as B but with anti-p62 and anti-α-tubulin antibodies. (**E**). SH-SY5Y cells were pre-treated with 1 µM epoxomicin for 4 h, prior to cell wash. Mock-DMSO or niclosamide treated cells were then further incubated for the indicated times and processed as in D.

Niclosamide has been shown to inhibit mTORC1, one of the two complexes that share the mTOR kinase [43]. We next sought to determine whether rapamycin, the prototypical mTORC1 inhibitor, could mimic the effect of niclosamide, as it was shown to induce autophagy [50]. Treatment with rapamycin alone did not induce any significant change in GFP-ubiquitin distribution in the cell (Fig. 6A) or in lysosome localization (Fig. S5A). Furthermore, in contrast to niclosamide, rapamycin did not prevent the accumulation of ubiquitinated proteins after the addition of MG132 (Fig. 6B). Levels of phosphorylated SK6 dropped in SH-SY5Y cells treated with rapamycin, confirming it was effectively inhibiting mTORC1 (Fig. 6B). Finally, we verified that rapamycin did not alter the formation of GFP-ubiquitin containing aggregates induced by proteasome inhibition (Fig. 6C). These results suggest that treatment of rapamycin alone is not sufficient to mimic the effects of niclosamide. Hence, mTORC1 inhibition by rapamycin does not induce the selective clearance of ubiquitinated proteins, and cannot prevent the formation of ubiquitin-containing aggregates caused by proteasome inhibition.

### Niclosamide affects p62 levels when the proteasome is inhibited

p62/Sequestosome 1 is a scaffold protein that tethers ubiquitinated proteins to autophagosomes, promoting their uptake to the lysosomes [28]. p62 is degraded in this process and up-regulation of autophagy causes a reduction of p62 levels [51]. In SH-SY5Y cells, both rapamycin and niclosamide induced a decrease of p62 levels, consistent with enhanced degradation by autophagy (Fig. S5B). Surprisingly, rapamycin did not cause any significant change of p62 levels when the proteasome was inhibited (Fig. 6D). It was previously shown that both mRNA and protein levels of p62 increase upon proteasome inhibition [52]. One possibility is that p62, which interacts with ubiquitinated proteins, becomes trapped in large aggregate structures caused by proteasome inhibition. As shown by the GFP-ubiquitin induced aggregate assay, these structures are not efficiently cleared after the induction of autophagy with rapamycin (Fig. 6C); hence, explaining why p62 levels remained high in the cells. In contrast, niclosamide, which prevents the formation of large ubiquitin-containing aggregates, caused a strong reduction of p62 during treatment with MG132 (Fig. 6D). To exclude that the decrease of p62 levels was due to the inhibition of p62 expression, we added niclosamide to cells after a pre-treatment of 4 h with the proteasome inhibitor epoxomicin in SH-SY5Y cells. Under these conditions, p62 levels continued to increase 2 and 4 h after the removal of epoxomicin, as proteasomes remained inactive in these cells (Fig. 6E). In contrast, when niclosamide was added after the pre-treatment, p62 levels decreased at 4 h (Fig. 6E). These results indicate that, in absence of proteasome activity, niclosamide induces the degradation of p62, most likely while it is associated with ubiquitinated proteins.

Selective autophagy pathways can specifically target to the lysosomes subsets of proteins or organelles like the ribosomes [53] or damaged mitochondria [54], [55], [56]. We examined whether niclosamide could induce the clearance of other specific subsets of proteins in addition to ubiquitin. We found that niclosamide did not induce any significant reduction of Rpl5 ribosome protein (Fig. S5C) or Tom20 mitochondria protein (Fig. S5D). These results indicate that niclosamide does not cause the clearance of other large complexes/organelles like ribosomes or mitochondria. We infer that niclosamide selectively prevents the accumulation of ubiquitinated proteins in the cell by possibly targeting them for degradation by the lysosomes (or via another proteasome-independent pathway), without targeting other large protein complexes.

## Discussion

We show here that inhibition of the proteasome induces the accumulation of poorly soluble ubiquitinated proteins associated with the formation of large cellular inclusions in the neuronal SH-SY5Y cells. In addition, GFP-ubiquitin in aggregates is not rapidly exchanged, indicating that the aggregating polypeptides conjugated to ubiquitin are steady in these structures. Notably, the formation of the ubiquitin-containing aggregates does not require the presence of any disease-specific proteins. A large portion of proteasome substrates is composed of short-lived misfolded or damaged proteins, which are mainly derived from abundant cellular proteins [2], [57]. Therefore, these aggregates are likely composed of a wide array of misfolded proteins accumulating during proteasome inhibition. In agreement with this, we found by mass spectrometry that chaperone proteins, which typically bind to misfolded proteins, are strongly enriched in these aggregates (I.B.W., M.B., J.M.W., T.M., manuscript submitted).

A decrease in proteasome activity has been observed during the formation of amyloid-like aggregates [58], [59]. It has been proposed that the ubiquitin-enriched inclusions may trap and stall the proteasome with irreversibly misfolded proteins [17]. Our results suggest that inhibition of the proteasome triggered by insoluble proteins in symptomatic cells would cause the accumulation of additional aggregation-prone proteins (i.e., common proteasome substrates) and most likely increase the stress in these cells. Remarkably, K63 of ubiquitin is required and is sufficient for the formation of these induced ubiquitin-containing aggregates. Proteasome inhibition has been shown to rapidly induce the accumulation of mostly K48-linked ubiquitin chains [6]. One possibility is that poly-ubiquitin chains are further ubiquitinated via K63 after prolonged proteasome inhibition to promote aggregate formation. Consistent with this idea the laboratory of J. Peng recently showed that K63-linkages accumulate in the cell after prolonged inhibition of the proteasome, but not as rapidly as K48 linkages [60]. There may be factors in the cell that might favour the “remodelling” of ubiquitin chains after prolonged proteasome inhibition to foster the aggregation of non-degraded proteasome substrates.

The autophagy-inducing compound niclosamide precludes the formation of the large ubiquitin-containing aggregates generated during proteasome inhibition. In the presence of niclosamide, there is no accumulation of poly-ubiquitinated proteins and a decrease of p62 during proteasome inhibition. In addition, niclosamide causes a redistribution of the lysosomes throughout the cell. It may therefore induce the clearance of ubiquitinated proteins and associated proteins by the lysosomes when the proteasome is inhibited. Surprisingly, we found that the induction of autophagy with the prototypical mTORC1 inhibitor rapamycin affected neither the formation of large GFP-ubiquitin containing aggregates, nor did it prevent the accumulation of poly-ubiquitinated conjugates caused by MG132. Rottlerin and perhexiline, which also induce autophagy by inhibiting mTORC1, similarly failed to alter the large aggregates induced by MG132. These results may indicate that up-regulation of autophagy by mTORC1 inhibition is not sufficient to induce a rapid clearance of large ubiquitin-containing aggregates. On the other hand, it has recently been discovered that rapamycin does not inhibit all functions of mTORC1 and that it does not strongly induce autophagosomes [61]. It is conceivable that mTORC1 is involved in this response and that niclosamide targets an mTORC1 substrate not inhibited by rapamycin.

Niclosamide induces a change of lysosome localization that may facilitate the uptake of ubiquitinated proteins when the proteasome is inhibited. In untreated cells, a majority of the soluble misfolded proteins is targeted for degradation by the ubiquitin proteasome system, while insoluble proteins are normally cleared by a cluster of lysosomes next to the centrosome [49](Fig S6A). Niclosamide causes a change of lysosome localization throughout the cell. It also causes the formation of small ubiquitin-containing inclusions next to the nucleus. Because lysosomes are redistributed throughout the cell, clearance of insoluble proteins next to the centrosome may be reduced, causing formation of small inclusions. When the proteasome is inhibited, ubiquitinated misfolded proteins accumulate in the cell and form large aggregate structures next to the centrosome (Fig. S6B). A small portion of ubiquitinated proteins is likely degraded by the lysosomes. For instance, we found that the addition of the lysosomal inhibitor chloroquine causes a slight increase in the aggregate size (data not shown). Intriguingly, the lysosomes are not able to clear the large ubiquitin-containing aggregates, even when autophagy is up-regulated with rapamycin. In contrast, the autophagy-inducing compound niclosamide blocks the accumulation of ubiquitinated proteins during proteasome inhibition. While still speculative, it is possible that this phenomenon is mediated by the degradation of ubiquitinated proteins by the lysosomes. An uptake of the ubiquitinated proteins could be favoured by the redistribution of the lysosomes before the formation of large aggregate structures (Fig. S6C). Our data suggest that particular cellular events (e.g., lysosome redistribution) may improve the efficacy of aggregate clearance.

## Materials and Methods

### Plasmids

The first ubiquitin sequence of the scUBI4 gene was amplified with 5′CTCAABCTTCTATGCAGATTTTCGTCAAGACT and 5′GAGCTCGAGACCACCTCTTAGCCTTAGCAC primers, and subcloned into pEGFP-C1 (Clonetech) using Hind III and Xho I restriction sites; the K48R and K63R were similarly amplified and subcloned into pEGFP-C1 using GST-ubiquitin cDNAs kindly provided by M. Petroski (Burnham Institute for Medical Research); the HA-ubiquitin constructs are described elsewhere [62].

### Cell culture

SH-SY5Y (ATCC, CRL-2266) cells were cultured in DMEM/F12 mixture (1∶1) supplemented with 10% FBS and 1% Pen/Strep (Invitrogen) at 37°C in 5% CO_2_. Calcium phosphate transfection was performed with cells at 30–60% confluency. Cells stably expressing GFP-ubiquitin fusion protein were selected with 0.5 mg/ml G418. At 70–90% cells confluency and 24 h after seeding on culture dishes or on HCl-treated coverslips, MG132 (DiscoveryScientific; 25 mg/ml in DMSO), epoxomicin (Biomol), clasto-lactacystin β-lactone (Biomol), nocodazole (Santa Cruz) and other indicated chemicals (Sigma) were added (with fresh media) at the indicated concentrations and for the indicated time. Lysotracker (Molecular Probes) was added 1 h prior to imaging.

### Microscopy, live cell imaging and FRAP

For fluorescence microscopy, cells were either fixed in −20°C methanol for 6 min or in 3% paraformaldehyde (PFA) for 15 min. All cells were incubated with Hoechst 33342 (Molecular Probes) and mounted on glass slides topped with ProLong Gold (Invitrogen). All images were acquired with an AxioObserverZ1 equipped with a Colibri-LED-system (365/470/590 nm) and an AxioCam HRm, and with either an 63x oil Plan-Apochromat (fixed cells) or an 40x oil EC Plan-Neofluar objective (live cells) prior to processing with the AxioVision rel 4.7 software (Zeiss). Wavelet extended focus function was used with all in-focused Z-stacked images for each picture. Live cell imaging was performed using the live cell system Chamlide IC (Life Cell Instrument). Cells were seeded on coverslips at 50% confluency, 24 h prior to the assembly of the 35 mm dish-type magnetic chamber and the addition of 1 ml of fresh media with MG132, which were then placed into the pre-warmed (37°C) microscope chamber supplied with 5% CO_2_. One hour after MG132 addition, 24 Z-stack images were taken (every 0.5 µm; each 25 ms) with the 470 nm LED module (set at 25% light intensity) every 15 minutes for 11 h. For FRAP analysis, fluorescence was bleached at a 488-nm (GFP) square region, and scanned images were collected at the indicated times on a Nikon SP1 confocal microscope. Fluorescence was quantified using Nikon EZview software. The remaining fluorescence in the bleached region after bleach was substracted from all data points. The ratio of the mean fluorescence in the bleached region over the pre-bleached value is shown for each data point.

### Analysis with automated fluorescence imager

20,000 cells/well were seeded on 96-well microtiter plates (Perkin-Elmer) 24 h prior to chemical treatment. Fresh medium supplemented with the indicated compounds was added for 8 h and cells were fixed with PFA prior to staining with 500 ng/ml 33342 Hoechst. Fixed cells were washed and kept in PBS containing 1 mM MgCl_2_ and 0.1 mM CaCl_2_ during the analysis with the Cellomics Arrayscan VTI Reader (Thermo Fisher) using a 20x objective. A cytoplasmic mask was generated using the compartment analysis algorithm and aggregates were detected using the “circ spot average intensity” or “circ spot average size” with a band pass fixed at 351 pixel intensity units for GFP signal (XF-100 filter). Objects with less than 10 average pixel intensity units (GFP) were automatically excluded from the analysis. 10 different 350 µm^2^ fields per well were analyzed, which typically resulted in >2000 analyzed cells/well. Means and standard deviations were calculated from data generated in three independent wells.

### Protein extracts

Cells harvested by trypsinization were lysed in 0.5% NP40, 150 mM NaCl, 25 mM Hepes pH 7.4, 1 mM PMSF, 1x complete protease inhibitors or RIPA buffer. All cell extracts were cleared at 16,000 g in a microfuge at 4°C for 10 minutes. High-speed centrifugation was performed at 166,000 g in a TLS-55 (Beckman) for 10 minutes. Normalization was performed after Bradford assays (Bio-Rad) prior to protein separation by 4–20% SDS-PAGE (Nusep inc). Immuno-staining were performed with P4G7 anti-ubiquitin (1∶250–1000, Santa Cruz), PSTAIR (1∶1000, Santa Cruz), (E-19)-R anti-α-tubulin (1∶1000, SantaCruz), C18 anti-S6K (1∶5000, Santa Cruz) and anti-p62 (1∶250, Santa Cruz) antibodies, followed by incubation with a secondary HRP-conjugated antibody (1∶3000, Bio-Rad).

## Supporting Information

Figure S1 Ubiquitin-containing aggregates caused by proteasome inhibition. (A) GFP-ubiquitin forms aggregates after proteasome inhibition with MG132. Transient calcium phosphate transfection was performed with SH-SY5Y cells seeded at 30–50% confluency with either GFP or GFP-ubiquitin cDNAs for 24 h prior to treatment with 5 µM MG132 or an according amount of DMSO for an additional 8 h. Cells were fixed with PFA and stained with Hoechst. Scale bar indicates 10 µm. (B) Histograms represent the averaged proportions of GFP-ubiquitin SH-SY5Y cells with none, one large GFP-ubiquitin inclusion alone, or one large inclusion with smaller inclusions, after treatment with 20 µM MG312 for 12 h, in two independent experiments (n = 100). (C) Immunofluorescence was performed on methanol fixed GFP-ubiquitin SH-SY5Y cells after the addition of 10 µM MG312 for 12 h. The FK2 (1∶250, Boston Biochem) antibody, which recognizes both mono and poly-ubiquitin, was employed in combination with Alexa 568 anti-mouse antibody (1∶1000, Invitrogen) and Hoechst staining. Scale bar represents 10 µm.
(1.86 MB TIF)

Figure S2 Ubiquitin-containing aggregates caused by proteasome inhibition localize at the centrosome. (A) GFP-ubiquitin aggregates localize at the centrosome. Immunofluorescence was performed on methanol fixed GFP-ubiquitin SH-SY5Y cells after treatment with 20 µM MG312 for 10 h. The anti γ-tubulin (1∶500, Sigma-Aldrich) antibody was employed in combination with Alexa 568 anti-mouse antibody and Hoechst staining. Scale bar represents 10 µm. (B) Nocodazole prevents the formation of large GFP-ubiquitin aggregates. Percentages of cells (n = 100) with at least one large inclusion (diameter >1.25 µm) were calculated in GFP-ubiquitin SH-SY5Y cells treated as indicated with 5 µM MG312, 2 µM nocodazole, and 1 µM clasto-lactacystin β-lactone for 8 h prior to methanol fixation and imaging.
(1.70 MB TIF)

Figure S3 Niclosamide affects the formation of the ubiquitin-enriched aggregates caused by proteasome inhibition. (A) Signal intensities of the GFP-ubiquitin aggregates (with standard deviations) in cells treated for 8 h with the indicated concentrations of niclosamide alone (light grey) or together with 5 µM MG132 (dark grey) were measured in three independent wells using the automated high-content fluorescence imager. (B) Fold decreases (with standard errors) of the aggregate signal intensity calculated using three series of niclosamide concentrations (same data as in A). (C) Niclosamide prevents the formation of the large ubiquitin-containing aggregates in presence of MG132. Representative images of cells incubated as indicated with 5 µM MG132 and 10 µM niclosamide prior to PFA fixation. Hoechst staining of the cells is shown in the insets. Scale bar represents 10 µm. The cells at the 8 h time point are the same as in Fig. 3B. (D) Niclosamide prevents the formation of the large ubiquitin-containing aggregates in cells pre-treated with epoxomicin. SH-SY5Y cells stably expressing GFP-ubiquitin were treated for 4 h with 1 µM epoxomicin before changing the media and adding DMSO or 10 µM niclosamide for another 4 h. Cells were fixed with cold methanol. Hoechst staining of the cells is shown in the insets. Scale bar represents 10 µm.
(1.86 MB TIF)

Figure S4 Niclosamide affects ubiquitination levels and lysosome distribution in the cell. (A) SH-SY5Y cells stably expressing GFP-ubiquitin were treated as indicated for 8 h. Equal amounts of proteins were separated by 4–20% SDS-PAGE followed by immunoblotting with anti-GFP (Roche) and anti α-tubulin (Sigma). (B) Niclosamide does not alter levels of histone H2A. SH-SY5Y cells were treated as indicated for 8 h and lysed in RIPA buffer. Equal amounts of proteins were separated by 4–20% SDS-PAGE followed by immunoblotting with histone H2A and PSTAIR antibodies. (C) Niclosamide causes a change of GFP-ubiquitin distribution in the cell. Representative images of cells incubated as indicated with DMSO or 10 µM niclosamide prior to PFA fixation. Hoechst staining of the cells is shown in the insets. Scale bar represents 10 µm. The cells at the 8 h time point were also shown in Fig. 4C. (D) Niclosamide causes a change of the lysosome distribution in the cell. Additional representative images (as shown in Fig. 4D) of cells incubated as indicated with DMSO or 10 µM niclosamide for 8 h. Lysotracker was added one hour prior to imaging of live cells performed in the pre-warmed Chamlide IC microscope chamber supplied with 5% CO2. Scale bar represents 10 µm.
(2.96 MB TIF)

Figure S5 Niclosamide but not rapamycin affects lysosome distribution and p62 levels during proteasome inhibition. (A) Representative GFP-ubiquitin SH-SY5Y cells treated with 20 nM rapamycin for 8 h, as well as with lysotracker for 1 h, prior to imaging of live cells. Scale bar represents 10 µm. (B–D) SH-SY5Y cells were treated as indicated for 8 h and lysed in RIPA buffer. Equal amounts of proteins were separated by 4–20% SDS-PAGE followed by immunoblotting with anti-p62 and PSTAIR antibodies (B), with anti-Rpl5 (molecular probes) and PSTAIR antibodies (C) and anti-Tom20 (1∶500, Santa Cruz) and alpha-tubulin antibodies (D; the same membrane as in Fig. 5B was used).
(2.08 MB TIF)

Figure S6 Schematic representations of the possible effects of niclosamide in the cell. (A) In unstressed cells, the majority of poly-ubiquitinated proteins are degraded by the proteasome. A small fraction of insoluble ubiquitinated proteins is targeted to the lysosomes, which are specifically enriched in one region of the cell (next to the centrosome). (B) The addition of the MG132 proteasome inhibitor leads to the accumulation of a large amount of non-degraded proteins that form a large aggregate. While a small portion of the proteins is degraded by the lysosomes, they cannot effectively clear all these proteins (even when autophagy is activated with rapamycin). (C) When niclosamide is added with MG132, the redistribution of the lysosomes throughout the cell may possibly facilitate the uptake and degradation of non-degraded ubiquitinated proteins prior to their accumulation in a large aggregate. This would lead to the formation of a small aggregate next to the nucleus as well as other smaller inclusions in the cell. Note that addition of niclosamide alone may only cause the degradation of a small portion of ubiquitinated proteins as the proteasome is still active. This could potentially lead to a small decrease of the amount of ubiquitin in the cell. Indeed, niclosamide treatment alone mainly affects histone H2A ubiquitination, which has been shown to be sensitive to proteotoxic stress and lower amount of free ubiquitin [32], [47]. In addition, because lysosomes are redistributed, the clearance of the insoluble proteins by the lysosome, accumulating next to the centrosome, may not be as effective as in unstressed cells, and a small amount of ubiquitinated proteins accumulates forming a small inclusion (next to the nucleus).
(0.15 MB TIF)

Movie S1 Live cell imaging of the formation of large ubiquitin-containing aggregates during proteasome inhibition. SH-SY5Y cells stably expressing GFP-ubiquitin were treated with 20 µM MG132 and place in the pre-warmed Chamlide IC microscope chamber supplied with 5% CO2. One hour later, imaging was initiated and images were taken every 15 minutes. Selected time points of this movie are shown in Fig. 1A.
(2.06 MB MOV)
