# Supplementary figures and images for: Niclosamide Prevents the Formation of Large Ubiquitin-Containing Aggregates Caused by Proteasome Inhibition
Source: PLoS One. 2010 Dec 23;5(12):e14410. doi: 10.1371/journal.pone.0014410 (PMC3009716; doi:10.1371/journal.pone.0014410)

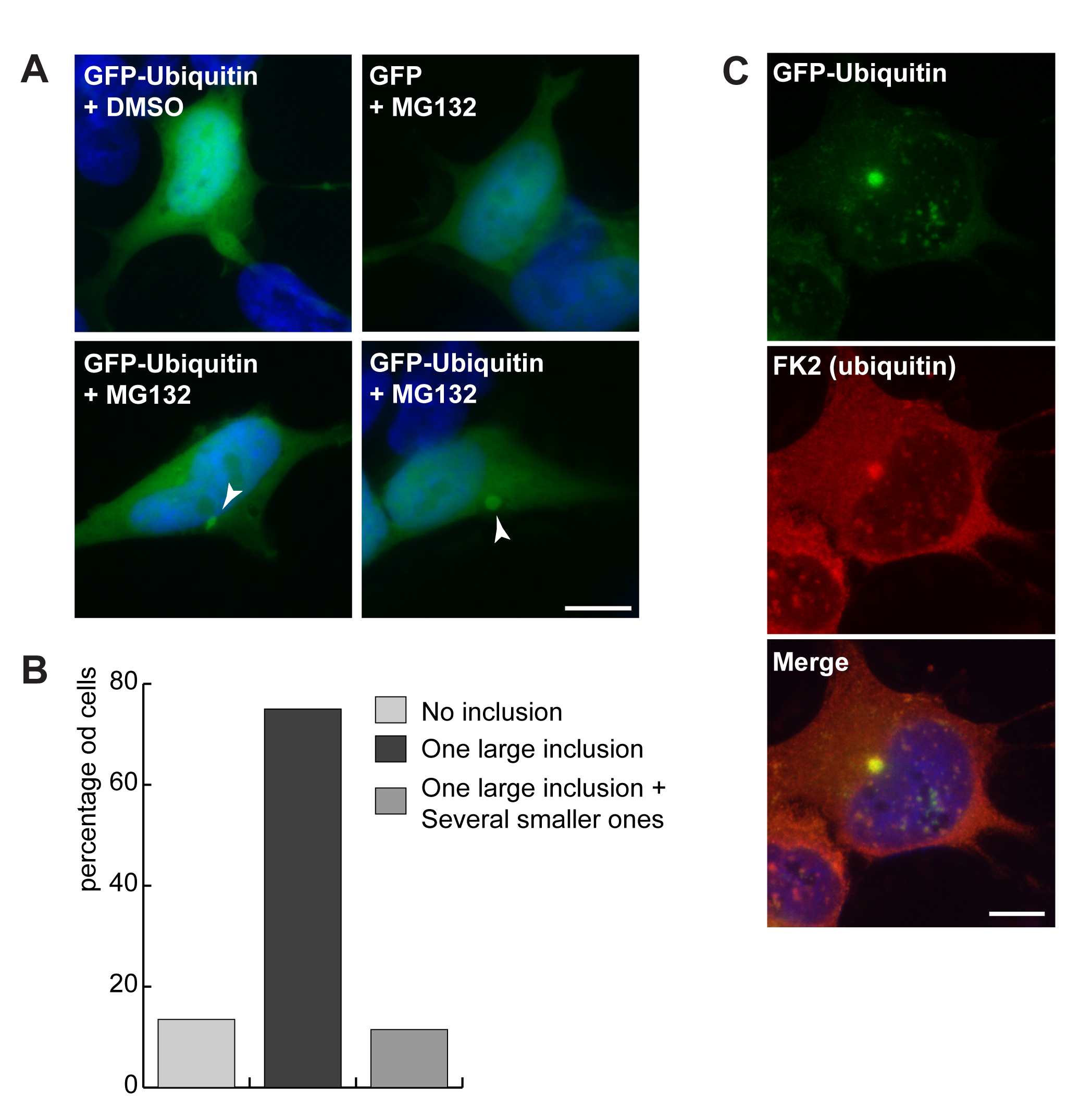

Supplement: Figure S1 — Ubiquitin-containing aggregates caused by proteasome inhibition. (A) GFP-ubiquitin forms aggregates after proteasome inhibition with MG132. Transient calcium phosphate transfection was performed with SH-SY5Y cells seeded at 30–50% confluency with either GFP or GFP-ubiquitin cDNAs for 24 h prior to treatment with 5 µM MG132 or an according amount of DMSO for an additional 8 h. Cells were fixed with PFA and stained with Hoechst. Scale bar indicates 10 µm. (B) Histograms represent the averaged proportions of GFP-ubiquitin SH-SY5Y cells with none, one large GFP-ubiquitin inclusion alone, or one large inclusion with smaller inclusions, after treatment with 20 µM MG312 for 12 h, in two independent experiments (n = 100). (C) Immunofluorescence was performed on methanol fixed GFP-ubiquitin SH-SY5Y cells after the addition of 10 µM MG312 for 12 h. The FK2 (1∶250, Boston Biochem) antibody, which recognizes both mono and poly-ubiquitin, was employed in combination with Alexa 568 anti-mouse antibody (1∶1000, Invitrogen) and Hoechst staining. Scale bar represents 10 µm. (1.86 MB TIF) [file pone.0014410.s001.tif]

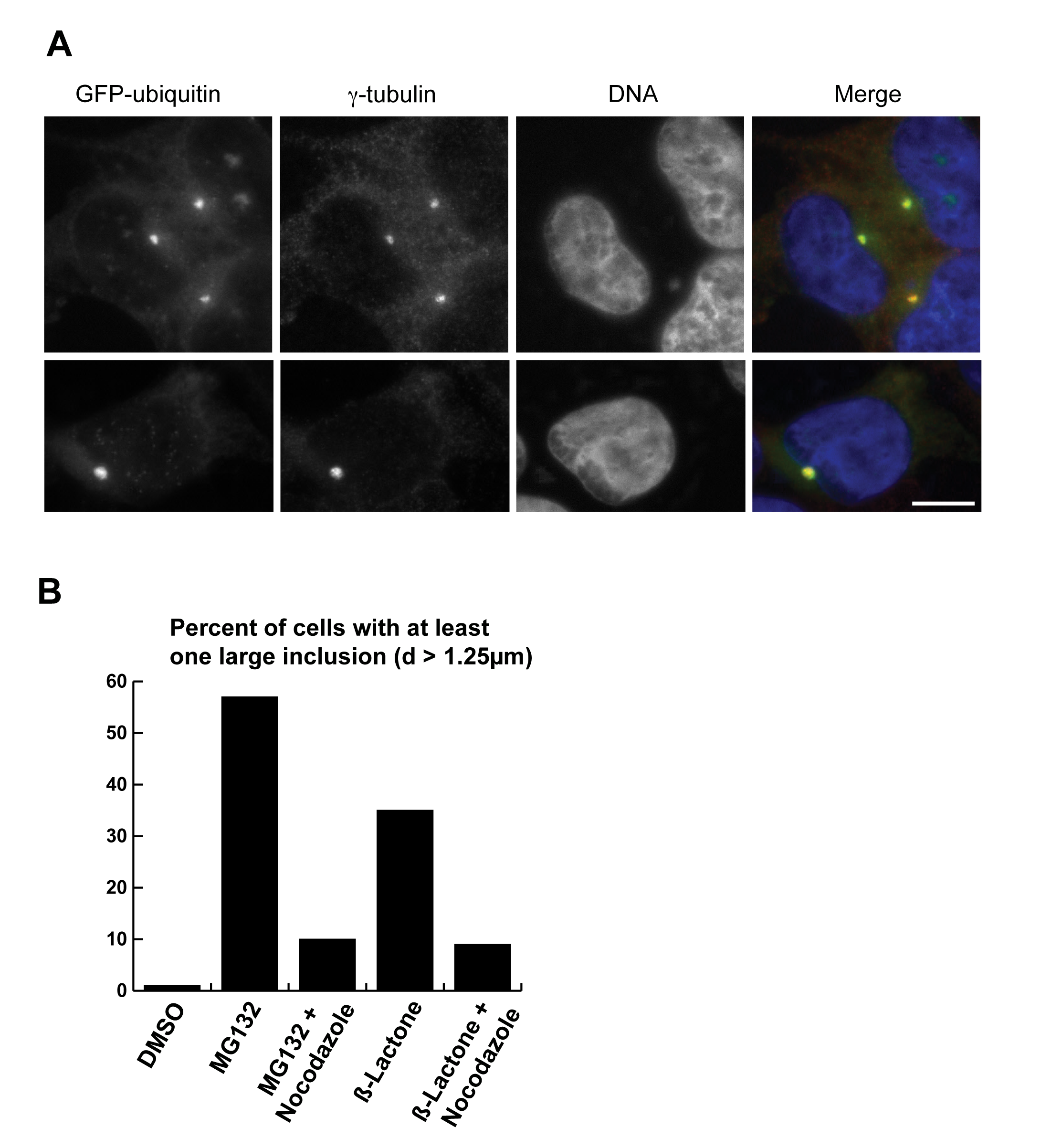

Supplement: Figure S2 — Ubiquitin-containing aggregates caused by proteasome inhibition localize at the centrosome. (A) GFP-ubiquitin aggregates localize at the centrosome. Immunofluorescence was performed on methanol fixed GFP-ubiquitin SH-SY5Y cells after treatment with 20 µM MG312 for 10 h. The anti γ-tubulin (1∶500, Sigma-Aldrich) antibody was employed in combination with Alexa 568 anti-mouse antibody and Hoechst staining. Scale bar represents 10 µm. (B) Nocodazole prevents the formation of large GFP-ubiquitin aggregates. Percentages of cells (n = 100) with at least one large inclusion (diameter >1.25 µm) were calculated in GFP-ubiquitin SH-SY5Y cells treated as indicated with 5 µM MG312, 2 µM nocodazole, and 1 µM clasto-lactacystin β-lactone for 8 h prior to methanol fixation and imaging. (1.70 MB TIF) [file pone.0014410.s002.tif]

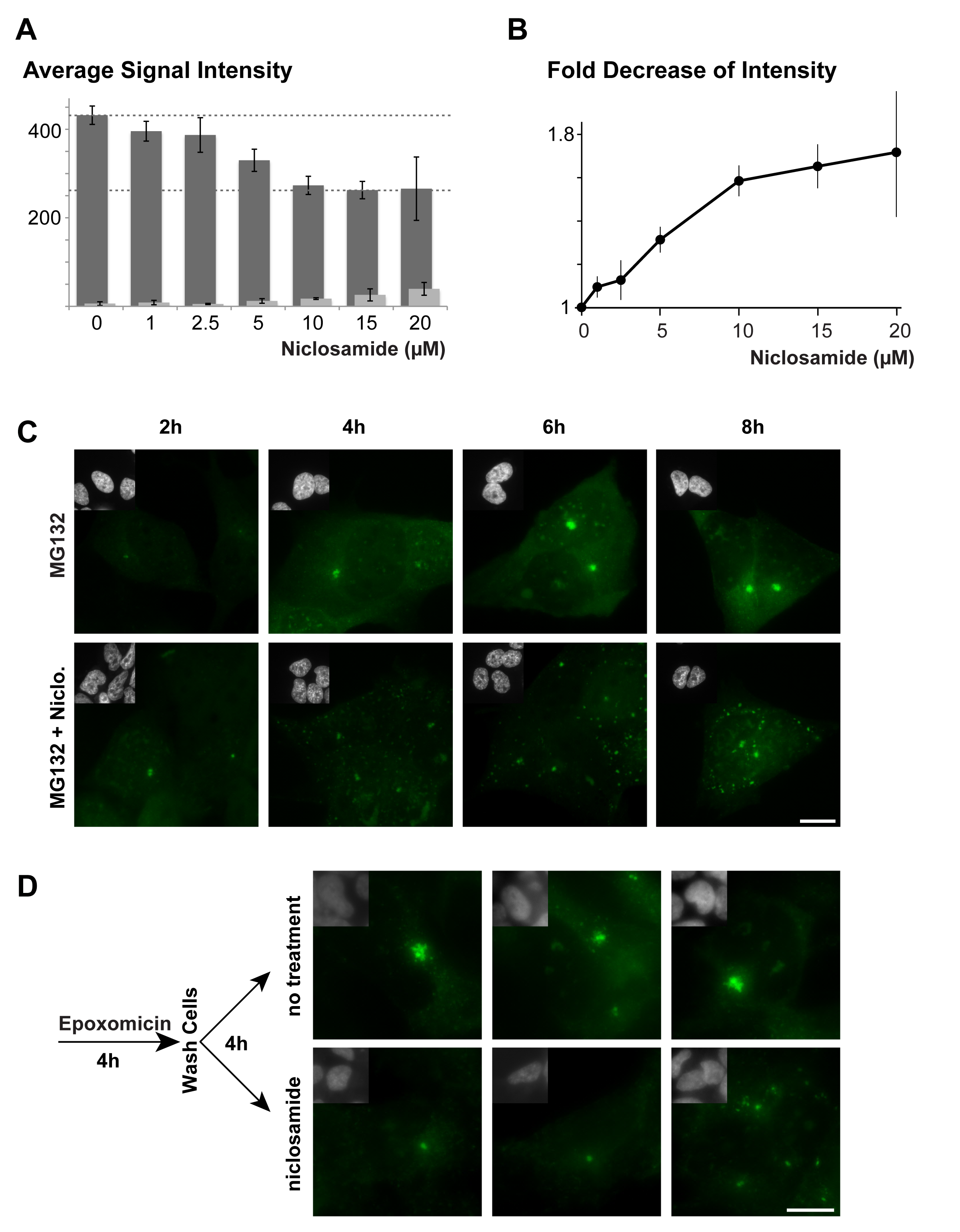

Supplement: Figure S3 — Niclosamide affects the formation of the ubiquitin-enriched aggregates caused by proteasome inhibition. (A) Signal intensities of the GFP-ubiquitin aggregates (with standard deviations) in cells treated for 8 h with the indicated concentrations of niclosamide alone (light grey) or together with 5 µM MG132 (dark grey) were measured in three independent wells using the automated high-content fluorescence imager. (B) Fold decreases (with standard errors) of the aggregate signal intensity calculated using three series of niclosamide concentrations (same data as in A). (C) Niclosamide prevents the formation of the large ubiquitin-containing aggregates in presence of MG132. Representative images of cells incubated as indicated with 5 µM MG132 and 10 µM niclosamide prior to PFA fixation. Hoechst staining of the cells is shown in the insets. Scale bar represents 10 µm. The cells at the 8 h time point are the same as in Fig. 3B. (D) Niclosamide prevents the formation of the large ubiquitin-containing aggregates in cells pre-treated with epoxomicin. SH-SY5Y cells stably expressing GFP-ubiquitin were treated for 4 h with 1 µM epoxomicin before changing the media and adding DMSO or 10 µM niclosamide for another 4 h. Cells were fixed with cold methanol. Hoechst staining of the cells is shown in the insets. Scale bar represents 10 µm. (1.86 MB TIF) [file pone.0014410.s003.tif]

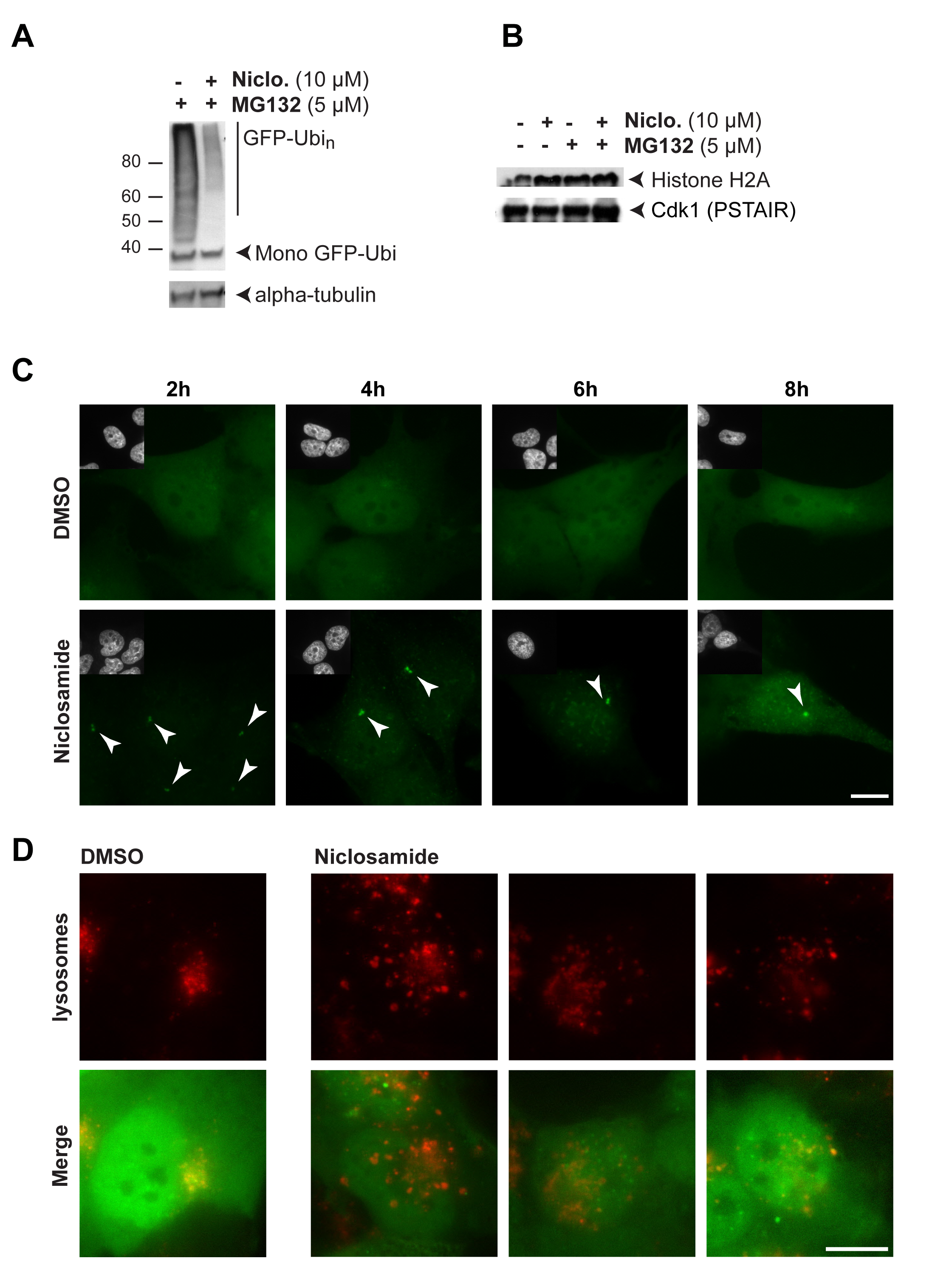

Supplement: Figure S4 — Niclosamide affects ubiquitination levels and lysosome distribution in the cell. (A) SH-SY5Y cells stably expressing GFP-ubiquitin were treated as indicated for 8 h. Equal amounts of proteins were separated by 4–20% SDS-PAGE followed by immunoblotting with anti-GFP (Roche) and anti α-tubulin (Sigma). (B) Niclosamide does not alter levels of histone H2A. SH-SY5Y cells were treated as indicated for 8 h and lysed in RIPA buffer. Equal amounts of proteins were separated by 4–20% SDS-PAGE followed by immunoblotting with histone H2A and PSTAIR antibodies. (C) Niclosamide causes a change of GFP-ubiquitin distribution in the cell. Representative images of cells incubated as indicated with DMSO or 10 µM niclosamide prior to PFA fixation. Hoechst staining of the cells is shown in the insets. Scale bar represents 10 µm. The cells at the 8 h time point were also shown in Fig. 4C. (D) Niclosamide causes a change of the lysosome distribution in the cell. Additional representative images (as shown in Fig. 4D) of cells incubated as indicated with DMSO or 10 µM niclosamide for 8 h. Lysotracker was added one hour prior to imaging of live cells performed in the pre-warmed Chamlide IC microscope chamber supplied with 5% CO2. Scale bar represents 10 µm. (2.96 MB TIF) [file pone.0014410.s004.tif]

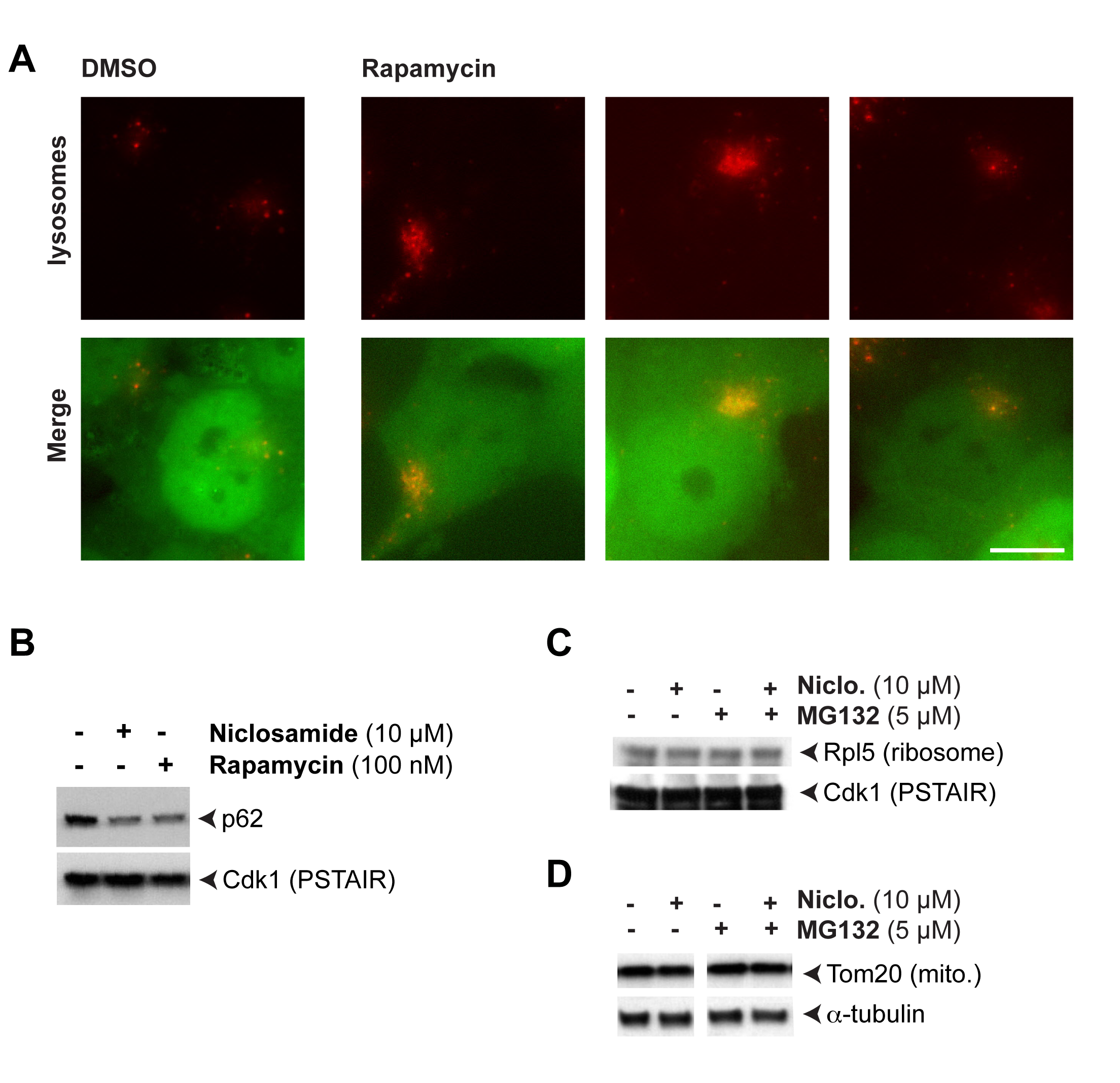

Supplement: Figure S5 — Niclosamide but not rapamycin affects lysosome distribution and p62 levels during proteasome inhibition. (A) Representative GFP-ubiquitin SH-SY5Y cells treated with 20 nM rapamycin for 8 h, as well as with lysotracker for 1 h, prior to imaging of live cells. Scale bar represents 10 µm. (B–D) SH-SY5Y cells were treated as indicated for 8 h and lysed in RIPA buffer. Equal amounts of proteins were separated by 4–20% SDS-PAGE followed by immunoblotting with anti-p62 and PSTAIR antibodies (B), with anti-Rpl5 (molecular probes) and PSTAIR antibodies (C) and anti-Tom20 (1∶500, Santa Cruz) and alpha-tubulin antibodies (D; the same membrane as in Fig. 5B was used). (2.08 MB TIF) [file pone.0014410.s005.tif]

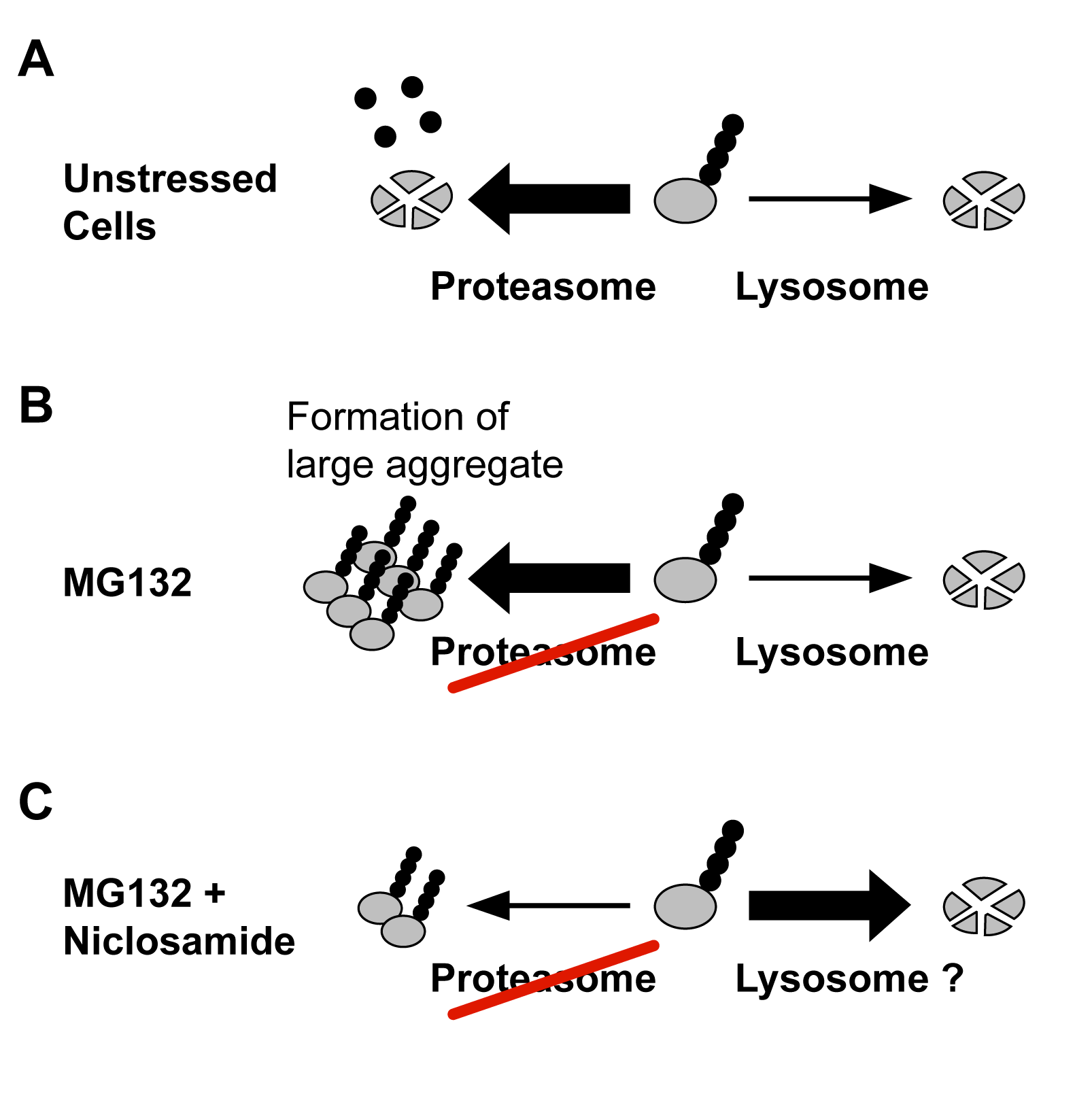

Supplement: Figure S6 — Schematic representations of the possible effects of niclosamide in the cell. (A) In unstressed cells, the majority of poly-ubiquitinated proteins are degraded by the proteasome. A small fraction of insoluble ubiquitinated proteins is targeted to the lysosomes, which are specifically enriched in one region of the cell (next to the centrosome). (B) The addition of the MG132 proteasome inhibitor leads to the accumulation of a large amount of non-degraded proteins that form a large aggregate. While a small portion of the proteins is degraded by the lysosomes, they cannot effectively clear all these proteins (even when autophagy is activated with rapamycin). (C) When niclosamide is added with MG132, the redistribution of the lysosomes throughout the cell may possibly facilitate the uptake and degradation of non-degraded ubiquitinated proteins prior to their accumulation in a large aggregate. This would lead to the formation of a small aggregate next to the nucleus as well as other smaller inclusions in the cell. Note that addition of niclosamide alone may only cause the degradation of a small portion of ubiquitinated proteins as the proteasome is still active. This could potentially lead to a small decrease of the amount of ubiquitin in the cell. Indeed, niclosamide treatment alone mainly affects histone H2A ubiquitination, which has been shown to be sensitive to proteotoxic stress and lower amount of free ubiquitin [32], [47]. In addition, because lysosomes are redistributed, the clearance of the insoluble proteins by the lysosome, accumulating next to the centrosome, may not be as effective as in unstressed cells, and a small amount of ubiquitinated proteins accumulates forming a small inclusion (next to the nucleus). (0.15 MB TIF) [file pone.0014410.s006.tif]
